# Synthesis and Characterization of Water-Soluble EDTA-Crosslinked Poly-β-Cyclodextrins Serving as Ion-Complexing Drug Carriers

**DOI:** 10.3390/ma19010207

**Published:** 2026-01-05

**Authors:** Zuzanna Podgórniak, Witold Musiał, Michał J. Kulus, Dominika Łacny, Aleksandra Budnik, Tomasz Urbaniak

**Affiliations:** 1 Department of Physical Chemistry and Biophysics, Pharmaceutical Faculty, Wroclaw Medical University, Borowska 211, 50-556 Wrocław, Poland; zuzanna.podgorniak@student.umw.edu.pl (Z.P.); lacnydominika@gmail.com (D.Ł.); tomasz.urbaniak@umw.edu.pl (T.U.); 2 Division of Ultrastructural Research, Wroclaw Medical University, Chałubińskiego 6a, 50-368 Wrocław, Poland; michal.kulus@umw.edu.pl

**Keywords:** polycyclodextrins, nanocarriers, ion sensitivity, drug delivery, polyelectrolyte complex, encapsulation

## Abstract

Water-soluble poly-β-cyclodextrins (PCDs), crosslinked with ethylenediaminetetraacetic acid dianhydride (EDTADA), were synthesized at varying β-CD:EDTADA molar ratios (1:6, 1:9, 1:12, 1:15) to develop multifunctional nanocarriers with the ability to complex drugs, polymers, and ions. All PCDs exhibited nanometric particle sizes (14 to 28 nm), negative zeta potential (−18 to −27 mV), and adjustable content of free carboxyl groups controlled by crosslinker ratio. Functional evaluations demonstrated effective Ca^2+^ chelation and a linear inclusion complexation profile with acyclovir, but not with naproxen, highlighting pH-dependent solubility effects. Additionally, PCDs successfully formed polyelectrolyte complexes with poly-L-lysine, indicating their potential as components of advanced drug delivery systems. Among the analyzed variants, PCD 1:6 showed reduced yields, fewer reactive groups, and diminished ion-binding capacity compared to formulations with higher crosslinker content. These findings underscore the importance of crosslinking density in modulating physicochemical and functional properties and support the potential of EDTA-crosslinked PCDs as versatile platforms for advanced, ion-sensitive biomedical applications.

## 1. Introduction

Cyclodextrins (CDs) are biocompatible, non-toxic, naturally occurring macrocyclic compounds—oligosaccharides composed of α-d-glucopyranose units linked by α-1,4-glycosidic bonds. The most common and widely used types are α-, β-, and γ-CDs, consisting of six, seven, and eight glucose units, respectively. Their toroidal cone-shaped structure features a hydrophilic outer surface due to the presence of hydroxyl groups and hydrophobic internal cavity capable of forming host–guest inclusion complexes. In the pharmaceutical industry, CDs are employed to mask unpleasant taste or odor, enhance the solubility and physicochemical stability of active pharmaceutical ingredients, and enable controlled drug release [1].

To broaden the range of CD applications, various polymerization methods have been developed, leading to the formation of polycyclodextrin (PCD) macromolecules. The solubility of PCD is highly influenced by the synthesis conditions, especially the type and ratio of the crosslinking agent. Lower crosslinker levels typically result in water-soluble or swellable materials, whereas increased crosslinking leads to the production of insoluble, rigid networks known as nanosponges. Epichlorohydrin, first used in 1965, is one of the most extensively studied crosslinking agents for PCD synthesis [2]. Since then, a wide range of alternative crosslinkers has been explored, including diisocyanates, polycarboxylic acids, dianhydrides such as pyromellitic dianhydride, diglycidyl ethers, and carbonyl-containing compounds like 1,1′-carbonyldiimidazole [3]. Due to the presence of interstitial cavities and a porous three-dimensional structure, crosslinked PCDs may exhibit an enhanced capacity to encapsulate guest molecules compared to native CDs. Furthermore, the polymeric network restricts the diffusion of encapsulated compounds, thereby enabling a more sustained and controlled release profile [4]. For this reason, they show promise in a range of biomedical applications, such as drug delivery systems for hyperuricemia [5], anticancer therapies [6], and antiepileptic treatments [7]. They have also been successfully used for environmental purposes [8,9] and in the food industry [10].

A particularly promising strategy involves the use of ethylenediaminetetraacetic acid (EDTA), as a crosslinker, which not only enables esterification of CD hydroxyl groups but also exhibits chelating properties toward divalent cations. Similarly, PMDA-based hyperbranched polymers showed the ability to interact with various metal cations, precipitating in the presence of selected divalent cations [11]. The key physicochemical properties of ion-interacting PCDs, such as surface charge or solubility, depends strongly on ionic environment. While these systems have been investigated for environmental purposes, e.g., removal of heavy metals and dyes [9], their potential in biomedical fields, especially as ion-sensitive drug carriers, remains underexplored. Variations in the ionic physiological environment and resulting disturbance of homeostasis are often linked to pathological conditions. Decreased iron levels may indicate anemia, while elevated calcium levels have been associated with cardiovascular diseases, skeletal disorders, inflammation at implantation sites or during wound healing. A variety of drug delivery systems exploiting described above ionic fluctuations have been developed—some designed to respond selectively to specific ions, while others exhibit non-specific ion sensitivity [12].

Sugammadex, the first clinically approved CD derivative that reverses neuromuscular blockade, exemplifies successful clinical application and highlights further development of CD derivatives as a promising route for novel therapeutics [13]. The encapsulation capacity of PCDs has been extensively investigated for a wide range of both hydrophilic and lipophilic drugs, such as 5-fluorouracil [14], doxorubicin [15], dexamethasone [16], itraconazole [17] and telmisartan [18]. EDTA-based nanosponges, which were previously designed for water filtration, can have a broader range of applications when synthesized using alternative approaches. For example, ibuprofen, a non-steroidal anti-inflammatory drug, was successfully encapsulated in β-CD/EDTA dianhydride-based nanosponges [19].

In this study, we aimed to synthesize EDTA-based PCDs characterized by aqueous solubility, which enhance their applicability as smart drug delivery system. Capability of such systems to complex both drugs and ions, along with their polyionic nature, indicates their possible role as components of polyelectrolyte systems, such as polyelectrolyte complexes or coatings obtained via layer-by-layer method.

## 2. Materials and Methods

### 2.1. Materials

Following chemicals were used in study: Disodium EDTA dihydrate, hydrochloric acid (35–38%), anhydrous triethylamine, anhydrous calcium chloride, diethyl ether, acetic anhydride, dimethyl sulfoxide, sodium hydroxide, and glacial acetic acid (99.5%), HPLC grade were purchased from Chempur (Piekary Śląskie, Poland). Ethanol (96%) was obtained from Stanlab (Lublin, Poland); pyridine, anhydrous from Eurochem BGD (Tarnów, Poland); β-Cyclodextrin from Pol-Aura (Morąg, Poland); PBS buffer, pH 7.4, 10 × concentrate from Stamar (Dąbrowa Górnicza, Poland); 1 M Tris-HCl buffer, pH 7.0 from EUR_X_ (Gdańsk, Poland); poly(L-lysine) hydrobromide (Mw ~50 kDa) from Polysciences Europe (Hirschberg, Germany); naproxen sodium salt (98% purity) from Aaron Chemicals (San Diego, CA, USA); phosphoric acid (85%) from Warchem (Zakręt, Poland); acetonitrile, HPLC grade from Avantor Performance Materials (Gliwice, Poland); deuterium oxide (≥99.9% deuteration) from Merck (Darmstadt, Germany); glutaraldehyde from Serva Electrophoresis (Heidelberg, Germany); Uranyless RTU and lead citrate from Electron Microscopy Sciences (Hatfield, PA, USA); acyclovir was kindly provided by Hasco-Lek (Wrocław, Poland).

### 2.2. Synthesis of EDTA-PCDs

Ethylenediaminetetraacetic acid dianhydride (EDTADA), used as a crosslinker, was synthesized by dehydrating disodium EDTA dihydrate (EDTA-Na). First, 20.079 g of EDTA-Na was dissolved in 200.0 mL of deionized water, and ~15.0 mL of concentrated HCl was added dropwise to induce precipitation. The precipitate was filtered, washed with 96% ethanol and diethyl ether (three times each), and dried at 105 °C for 2 h. The dried EDTA was suspended in anhydrous pyridine (45.0 mL), and acetic anhydride (40.0 mL) was added. The mixture was stirred at 65 °C for 24 h, then filtered, washed three times with acetic anhydride and diethyl ether, dried, and stored in a desiccator.

Polymerization was carried out at room temperature using anhydrous reagents to prevent EDTADA hydrolysis. β-CD was dried in a moisture analyzer at 105 °C until constant weight before use. Reactions were performed at β-CD:EDTADA molar ratios of 1:6, 1:9, 1:12, and 1:15. The monomer and crosslinker were dissolved in anhydrous DMSO (10 mL) in glass vials, yielding final β-CD concentrations of 0.043 M and EDTADA concentrations of 0.259 M, 0.388 M, 0.517 M, and 0.646 M for the respective ratios. After stirring for 1 h to fully dissolve β-CD, triethylamine (TEA, 0.5 mL) was added, and the reaction proceeded for 24 h. Polymerization was terminated by adding deionized water (10 mL). The reaction products were purified by dialysis (Spectra/Por 1, MWCO 6–8 kDa) against deionized water (700 mL), with conductivity monitored to ensure removal of unreacted reagents. Dialysis continued for 19 days with eight water changes, after which the products were lyophilized. The polymerization yield was determined gravimetrically by weighing the purified, dried PCDs and calculating based on the initial mass of the substrates.

### 2.3. Conductometric Titration

The non-esterified carboxyl group content of the PCDs was determined by conductometric titration using a TitroLine^®^ 7800 titrator coupled with a CC-511 conductometer and an EC-210 conductivity sensor (both from Elmetron, Zabrze, Poland). A 4.61 mM HCl solution was used as the titrant. Each PCD sample (20.0 mg) was dissolved in deionized water (50 mL), and 1.5 mL of 0.1 M NaOH was added (1.75 mL for samples prepared at a 1:15 β-CD:EDTADA ratio). The titrant was added at 0.5 mL/min to a total volume of 40.0 mL. Measurements were carried out at 21 ± 0.5 °C under continuous magnetic stirring. All titrations were performed in triplicate, and results were reported as mean ± standard deviation (SD).

### 2.4. Fourier Transform Infrared Spectroscopy

Fourier-transform infrared spectroscopy (FTIR) analysis was applied to confirm the formation of polymer networks from native CDs. The spectra of the obtained PCDs and β-CD were recorded using a FTIR spectrophotometer with an ATR accessory (Nicolet iS50, Thermo Fisher Scientific, Waltham, MA, USA). A total of 32 scans were collected for each sample in the range of 4000–400 cm^−1^. Before each measurement, a background spectrum was recorded and subsequently subtracted from the sample spectra.

### 2.5. Measurement of Hydrodynamic Diameter and Zeta Potential

The hydrodynamic diameter, polydispersity index (PDI), and zeta potential of the PCD were analyzed using a Zetasizer Nano ZS ZEN3600 (Malvern Panalytical Ltd., Malvern, UK). Each PCD variant was analyzed at 2.5 mg/mL in PBS. Zeta potential in 3 replicates (polycarbonate cuvettes with built-in copper electrodes) and hydrodynamic diameter in 4 replicates (transparent polystyrene cuvettes) were measured at 25 ± 0.1 °C. Results were reported as mean ± SD.

The interaction of PCDs with calcium ions was evaluated by titration of PCD solutions (2.5 mg/mL in 0.1 M Tris-HCl) with 0.1 M CaCl_2_ prepared in the same buffer. The titrant was added incrementally until a final Ca^2+^-to-PCD mass ratio of 13.0 µmol/mg was reached. Hydrodynamic diameter values were averaged from at least four measurements, and zeta potential values from three measurements.

To assess the effect of polycation addition on PCD particle size and zeta potential, PCD 1:12 was dissolved in PBS (pH 7.4) at 1.0 mg/mL. Poly-L-lysine (PLL, 5.0 mg/mL in PBS) served as the polycation. Hydrodynamic diameter and zeta potential were first measured for PCD alone, after which PLL aliquots were incrementally added to the cuvette, achieving PLL amino group to PCD carboxyl group molar ratios from 0.00 to 1.99. Mean values were obtained from at least three measurements for each condition.

### 2.6. Potentiometric Titration

The measurements were performed using a TitroLine 7800 dosing system with a CC-511 potentiometer (Elmetron, Zabrze, Poland). A Ca^2+^-selective glass electrode (Monokrystaly, Turnov, Czech Republic) served as the indicator, with an Ag/AgCl reference electrode (RAE 111B, Monokrystaly, Prepere, Czech Republic). Each PCD variant (20.0 mg) was dissolved in deionized water (50 mL), and 1.0 mL of 0.1 M NaOH was added to adjust pH to ~11.0. A 0.005 M CaCl_2_ solution was titrated in at 0.5 mL/min to a total of 20.0 mL, with continuous magnetic stirring maintained throughout.

### 2.7. Phase Solubility

Phase solubility studies for NAP and acyclovir (ACV) were performed using the Higuchi and Connors method [20]. Excess drug (25 mg ACV or 7.5 mg NAP) was added to test tubes containing 5 mL of 1: 12 or 1:15 PCD solutions in PBS (pH 7.4) at concentrations ranging from 0.0 to 12.0 mg/mL. NAP was obtained by acidifying its sodium salt with concentrated HCl. Suspensions were sonicated for 5 min, then shaken at 200 rpm at room temperature for 72 h to reach equilibrium. After centrifugation (15,000 rpm, 15 min), supernatants were diluted as needed, and drug concentrations were quantified by HPLC. Phase solubility diagrams were constructed by plotting dissolved drug concentration against PCD concentration.

### 2.8. High-Performance Liquid Chromatography

Quantitative determination of NAP and ACV was performed using HPLC on a Hitachi Primaide HPLC system (Hitachi HTA, Schaumburg, IL, USA) equipped with a Primaide 1410 UV detector.

For ACV, chromatographic separation was carried out at 25 °C using a mobile phase consisting of a 100% aqueous solution of 0.1% (*v*/*v*) triethylamine, with the pH adjusted to 2.5 using 85% ortho-phosphoric acid. Separation was achieved on a LiChrospher 100 RP-8e column (5 µm; 4 mm × 250 mm, Merck Millipore, Burlington, MA, USA) at a flow rate of 1.0 mL/min. The injection volume was 10 µL, and detection was performed at a wavelength of 255 nm.

For NAP, separation was conducted on an Agilent ZORBAX Eclipse XDB-C8 column (5 µm; 4.6 mm × 150 mm, Agilent Technologies, Santa Clara, CA, USA) at 25 °C. The mobile phase consisted of acetonitrile, water, and glacial acetic acid in a ratio of 450:540:10 (*v*/*v*/*v*), and the flow rate was maintained at 1.0 mL/min. An injection volume of 20 µL was applied; the UV detector operated at 254 nm.

Drug concentrations were determined using the external standard method. Linear calibration curves were obtained in the concentration ranges of 1.5625–200.0 µg/mL for ACV and 5.0–150.0 µg/mL for NAP, with correlation coefficients (r^2^) of 0.999 for both compounds.

### 2.9. Transmission Electron Microscopy

PCD samples were visualized using a transmission electron microscopy (TEM) with a modified protocol for exosome staining [21]. First, Formvar-carbon-coated copper grids (Thermo Scientific, Waltham, MA, USA) were pretreated with UV irradiation in ozone cleaner for 10 min (Ossila, Sheffield, United Kingdom) and then treated with 0.05% (*w*/*v*) PLL solution in water (5 min). The grids were placed on 10 µL drops of the sample for 60 min. Then, the grids were washed by placing 30 µL drops of PBS buffer on them three times for two minutes each. Then, the grids were fixed in a 2% paraformaldehyde in PBS solution for 10 min, washed and post-fixed in a 2.5% glutaraldehyde solution. Next, the grids were washed in Milli-Q water (Merck Millipore, Darmstadt, Germany), five times for two minutes each and counterstained with Uranyless RTU solution and a 3% lead citrate solution. The dried grids were examined using a JEM-1011 transmission electron microscope (JEOL, Tokyo, Japan) at an accelerating voltage of 80 kV.

### 2.10. ^1^H Nuclear Magnetic Resonance Spectroscopy Measurements

^1^H NMR spectra were recorded using a water-suppression pulse sequence (zgesgp) on a Bruker AVANCE III 600 MHz spectrometer equipped with an Ascend™ magnet (Bruker BioSpin, Billerica, MA, USA). Samples were analyzed in D_2_O at 25 °C. Acquisition parameters were: spectral width 10 ppm, 8 scans, relaxation delay 1 s, and acquisition time 2.73 s. Chemical shifts are reported in ppm and referenced to the residual HOD peak at 4.79 ppm. Data were processed using TopSpin 4.3.0 software.

Samples included β-CD, PCD 1:12, and PCD 1:15 (9 mg/mL) as well as EDTA-Na, PCD 1:6, and PCD 1:9 (15 mg/mL). For hydrolysis experiments, a small amount of NaOH was added to PCD 1:15 several hours before measurement. For TEA–PCD interaction studies, TEA was added at 10 mg/mL, and β-CD and EDTA-Na were also combined with TEA at a 1:1 molar ratio.

### 2.11. Chemical Composition Analysis

The elemental composition of PCD variants (1:6, 1:9, 1:12, and 1:15) was analyzed by Energy Dispersive X-ray Spectroscopy (EDS) using a Thermo Scientific Phenom ProX desktop SEM (Thermo Fisher Scientific, Waltham, MA, USA). At least four representative surface areas were mapped per sample to assess local elemental distribution. Spectra from these areas were used to calculate average weight percentages of detected elements. Measurements were performed with a Silicon Drift Detector (SDD) at 15 kV, with a 60 s acquisition time per area. Standardless quantification employed ZAF correction via the instrument’s Element Identification (EID) software (v. 2.0.1-rel).

## 3. Results and Discussion

### 3.1. Elemental Analysis and Synthesis

The reaction yields of conducted polymerization process differed between employed variants, with notably higher yields observed in reactions performed with lower monomer to crosslinker ratio. A decrease in the molar ratio of β-CD to crosslinker resulted in a shift in the reaction equilibrium toward product formation, leading to higher polymerization yields (Table 1).

A similar trend and comparable yields were previously reported using β-CD and diphenyl carbonate as the crosslinking agent [5]. The difficulty in achieving a higher yield may be attributed to low molecular weight products loss during purification, as well as to the possible inhibition of the crosslinking process at a certain stage of the reaction. Increasing EDTADA content promotes formation of more densely branched EDTA-rich polymer networks. As branching progresses, steric crowding around β-CD hydroxyls limits further propagation, yielding larger, water-soluble macromolecular species that remain above the dialysis cutoff and increase the isolated yield. At higher crosslinker content, the polymerization reaches this structural limit earlier, producing smaller oligomers (below 7–8 kDa) that are removed during purification. Semiquantitative elemental analysis confirmed the presence of carbon, oxygen, and nitrogen in the polymer network, with ratios reflecting theoretical ratios derived from reaction mixture composition. Increasing N and O contents with higher EDTA content, reflecting a greater incorporation of EDTA-derived functional groups. A fraction of the nitrogen also originates from triethylammonium counterions bound to EDTA carboxylates, but both species track with EDTA abundance; thus, higher %N corresponds to a higher density of acidic/carboxylate groups in the polymer. These compositional differences correlate with functional behavior: EDTA-rich PCDs exhibit stronger Ca^2+^ binding and polyelectrolyte interactions and form slightly larger, more branched nanogels, whereas the low-EDTA formulation shows minimal ion binding and weaker interaction capacity.

### 3.2. FTIR Analysis

**Figure 1:**
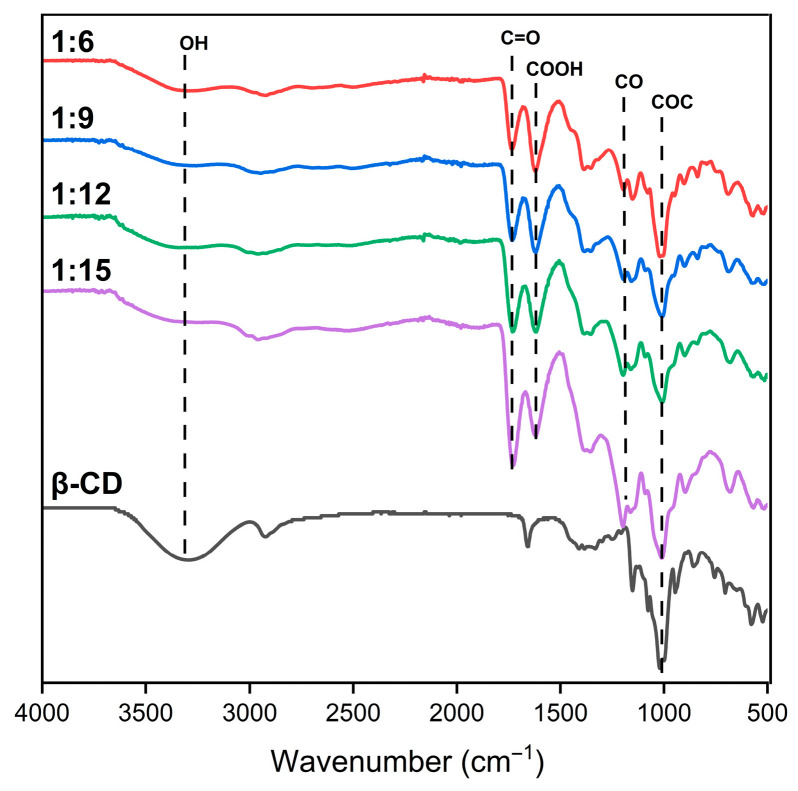
FTIR spectra of β-CD polycondensates obtained using monomer-to-crosslinker ratios of 1:6, 1:9, 1:12, and 1:15, along with pure β-CD.

FTIR spectra were analyzed for characteristic absorption bands indicating ester bonds and free carboxyl groups derived from EDTA. These groups are capable of dissociation, imparting polyanionic character to the synthesized carriers. In the spectrum of native β-CD a broad band between 3000 and 3600 cm^−1^ was observed, corresponding to the O–H stretching vibrations of primary hydroxyl groups. In contrast, a marked reduction in this band in the spectra of the polymerized products suggests the involvement of hydroxyl groups in ester bond formation (Figure 1).

Additionally, a characteristic peak at 1020 cm^−1^ was identified for both β-CD and the various PCD samples, attributed to the stretching vibrations of the glycosidic bonds present in the CD ring structure, as reported in previous studies [22]. Across all PCD variants, a distinct peak near 1730 cm^−1^ was detected, assigned to the C=O stretching vibrations of ester groups. The intensity of this peak increased with a lower molar ratio of β-CD to crosslinking agent, indicating a greater degree of crosslinking at elevated crosslinker concentrations. A comparable trend was observed for the absorption band around 1200 cm^−1^, attributed to the formation of additional C–O bonds within the polymer structure, which is consistent with similar results previously reported in the literature [6,8,9]. The FTIR spectrum of EDTA shows an intense peak at 1620 cm^−1^, corresponding to the presence of carboxyl groups [9]. The appearance of a analogous band in the spectra of all synthesized PCDs indicates the presence of residual free carboxyl groups, which contribute to the polyanionic character of the resulting macromolecules. In summary, FTIR spectra demonstrate intact CD glycosidic units (~1020 cm^−1^), newly formed ester bonds (C=O ~1730 cm^−1^; C–O ~1200 cm^−1^), and residual EDTA-derived carboxyl groups (~1620 cm^−1^). Ester band intensity increases with EDTADA content, reflecting higher crosslinking density, while the free carboxylates account for the polyanionic charge of the PCDs.

### 3.3. ^1^H NMR Spectral Data

**Figure 2:**
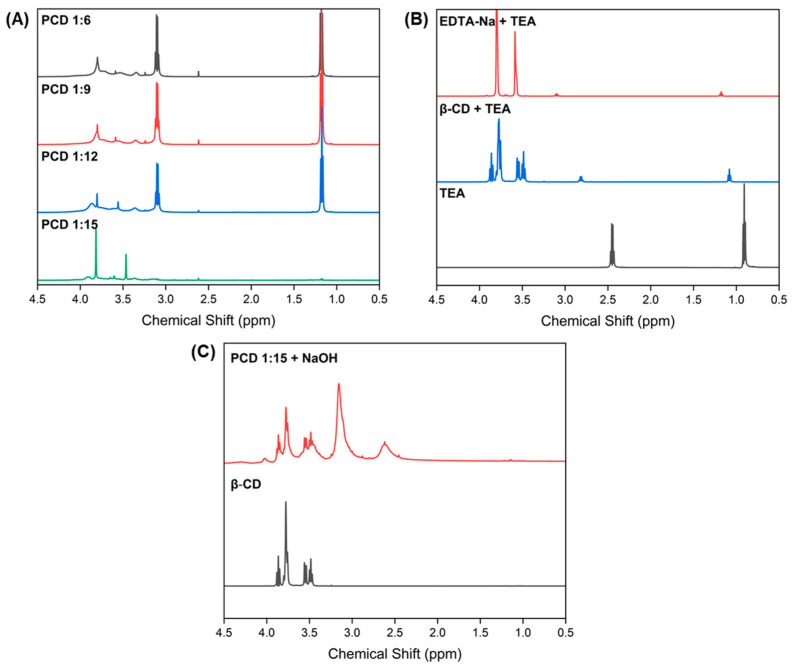
^1^H NMR spectra (600 MHz, D_2_O) of (**A**) PCD 1:6, PCD 1:9, PCD 1:12, and PCD 1:15; (**B**) EDTA-Na-TEA, β-CD-TEA (both in a 1:1 molar ratio), and pure TEA; (**C**) PCD 1:15 after hydrolysis with NaOH, and pure β-CD.

^1^H NMR spectroscopy was performed to verify the structures of the obtained PCDs. In all PCD variants, two sharp singlets were observed at 3.80 ppm and 3.58 ppm, corresponding to methylene protons from the acetate group and methylene protons from the EDTA backbone, respectively (Figure 2A) [23].

In the PCD spectra, typical β-CD signals were absent, which might be linked to not sufficiently large transverse relaxation time of β-CD protons entrapped in crosslinked, gel like network—the phenomenon observed for polysaccharides and supramolecular structures [24,25]. To confirm this hypothesis, excess NaOH was added to the PCD 1:15 variant; following hydrolysis, characteristic β-CD peaks reappeared in the spectrum (Figure 2C).

The presence of EDTA signals in the PCD spectra indirectly supports the success of the polymerization process and suggests that these moieties have greater spatial freedom, likely due to their localization on the PCD surface. A triplet at 1.20–1.15 ppm and quartet at 3.12–3.07 ppm were assigned to methyl protons and methylene groups of a TEA, which was used in the polymerization process (Figure 2A). Compared to the spectrum of pure TEA (Figure 2B), the PCD spectra exhibit a downfield shift of the TEA signals, indicating protonation and salt formation [26] (Figure 2B). Free TEA shows resonances at 0.90 ppm (CH_3_) and 2.45 ppm (CH_2_). In the presence of β-CD, only minor shifts are observed (1.08 and 2.82 ppm), indicating weak nonspecific interactions. However, mixing TEA with disodium EDTA-Na results in pronounced downfield shifts to 1.18 and 3.11 ppm, characteristic of protonated triethylammonium (TEA·H^+^), consistent with acid–base exchange involving the remaining acidic groups of EDTA salt. The PCDs spectra shows nearly identical TEA signals (1.19 and 3.12 ppm), demonstrating that TEA remains in the polymer as TEA·H^+^ electrostatically paired with EDTA-derived carboxylates. The persistence of TEA after extended dialysis supports that it is not present as free TEA but as a bound counterion within the polyanionic network.

### 3.4. PCD Morphology and Structure

DLS analysis was performed to evaluate the particle size and size distribution of the synthesized PCDs. These are critical parameters impacting drug delivery process and describing the homogeneity and stability of the colloidal system. The average hydrodynamic diameters of PCD particles obtained at different molar ratios of reagents were as follows: 1:6—13.93 ± 0.76 nm; 1:9—26.78 ± 0.51 nm; 1:12—22.38 ± 0.37 nm; 1:15—27.78 ± 0.59 nm. PCD-based nanocarriers with diameters below 100 nm have previously been described in the context of biomedical applications (Figures S1–S4) [27,28]. In this study, the smallest particle size was obtained with the lowest amount of crosslinking agent, while the largest particles were observed with the highest amount of crosslinker. However, observed differences were minor and no relationship was observed with a decreasing monomer-to-crosslinker molar ratio. The PDI values for the tested samples were as follows: 1:6—0.330; 1:9—0.245; 1:12—0.361; 1:15—0.275. These results indicate some variability in particle size, corresponding to a moderately polydisperse particle size distribution. All formulations showed moderately low polydispersity without a clear trend, indicating that each PCD sample forms a relatively uniform nanogel population in the tens of nanometers. The size did not increase indefinitely with crosslinker, suggesting that excess EDTA leads to more branching rather than growth beyond ~30 nm. According to previous works, β-CD:EDTA molar ratio exceeding 1:6 leads to predominantly β-CD branching, instead of crosslinking, which would lead to formation of large hydrogel networks [29]. In line with this observation, HNMR analysis revealed a high surface concentration of EDTA, and the crosslinking-driven growth of PCDs in this study was limited to approximately 28 nm.

**Figure 3:**
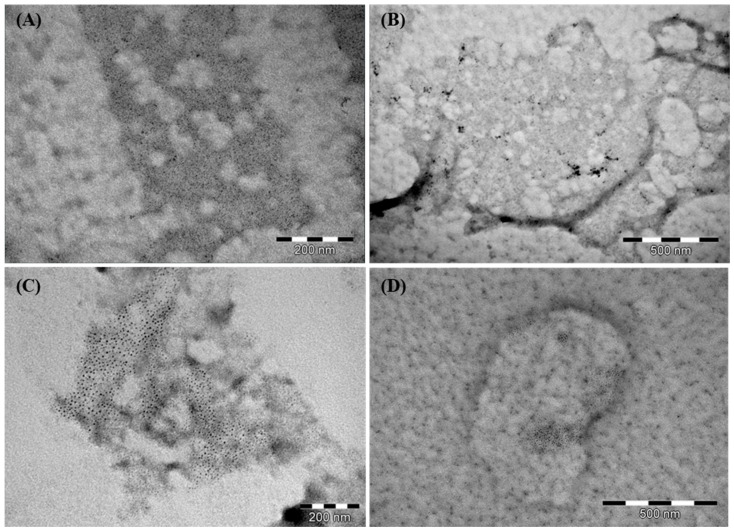
TEM image of PCDs adsorbed onto a positively charged PLL layer: (**A**) PCD 1:6, (**B**) PCD 1:9, (**C**) PCD 1:12, and (**D**) PCD 1:15.

Prior to TEM imaging, the hydrophobic Formvar surface was first hydrophilized and then coated with the positively charged polyelectrolyte PLL. This step was essential to retain the PCD nanoparticles on the grid during rinsing and staining. The PLL layer provided a positively charged surface that effectively captured the anionic PCDs and enhanced image contrast. In the TEM images (Figure 3), PLL-coated regions appear decorated with dark, near-spherical particle clusters, most prominently for the sample prepared at a β-CD:EDTA molar ratio of 1:12. Because the PCD nanogels are highly hydrated in aqueous conditions, they are prone to collapse and partial aggregation during drying. As a result, the TEM images show clustered electron-dense regions rather than individually resolved nanoparticles, despite the nanoscale (~20 nm) particle size determined in solution. The contrast between the PLL layer and the adsorbed PCDs results from the differing affinities of the positively charged dye used for staining. The nanometric size of the observed structures was consistent with DLS measurements, further confirming the formation of nanoscale assemblies. It is important to note that the hydrodynamic diameters obtained from DLS reflect the swollen particles in solution, including their ionic double layers, and are typically larger than the sizes of the dried particles visualized under vacuum in TEM.

### 3.5. Ionic and Complexing Properties

The ability of the investigated nanoparticles to interact with cationic entities via free carboxyl groups enables their incorporation into more complex polyelectrolyte composites and ion-interacting systems. This capability was examined using zeta potential measurements, conductometric titration and potentiometric titration (Table 2).

Zeta potential measurements ranged from −18.38 ± 2.45 for PCD 1:6 to −27.38 ± 2.51 mV for PCD 1:15, indicating good colloidal stability, due to electrostatic repulsion. The negative values reflect the presence of EDTA-derived free carboxyl groups, which primarily contribute to the polyanionic character of the synthesized PCDs. The greatest decrease in the zeta potential value was observed between the PCD 1:6 and PCD 1:9 samples, with less pronounced changes noted with further decrease β-CD–crosslinker ratio. According to previous findings, at a molar ratio of 1:6, the process of effective crosslinking predominates, and the surface of individual cyclodextrin molecules is not fully saturated with crosslinking agent, leaving free sites available for the attachment of additional EDTA molecules. With a higher availability of the crosslinker, branching of cyclodextrin units predominates. However, due to the steric constraints on particle surface, the density of the branching groups increases only slightly, resulting in minimal changes in the analyzed zeta potential values.

Conductometric titration enabled the quantification of unreacted carboxyl groups, showing a progressive increase in the amount of titrant required for the titration of the sodium salt of PCD at lower β-CD:EDTADA molar ratios. This trend indicates that a higher concentration of free carboxyl groups in polymer networks may be obtained with elevated EDTADA content. These findings are crucial for evaluating the suitability of the prepared nanocarriers for divalent cation complexation and the formation of polyelectrolyte complexes. The results are consistent with the earlier considerations, that branching of β-CD dominates over effective crosslinking, at β-CD–crosslinker molar ratios exceeding 1:6, leading to a higher number of unbound carboxyl groups. Carboxyl content increases as the β-CD–crosslinker ratio decreases, while the zeta potential remains unchanged, suggesting that the additional carboxyl groups are internal and do not influence the measured surface charge.

**Figure 4:**
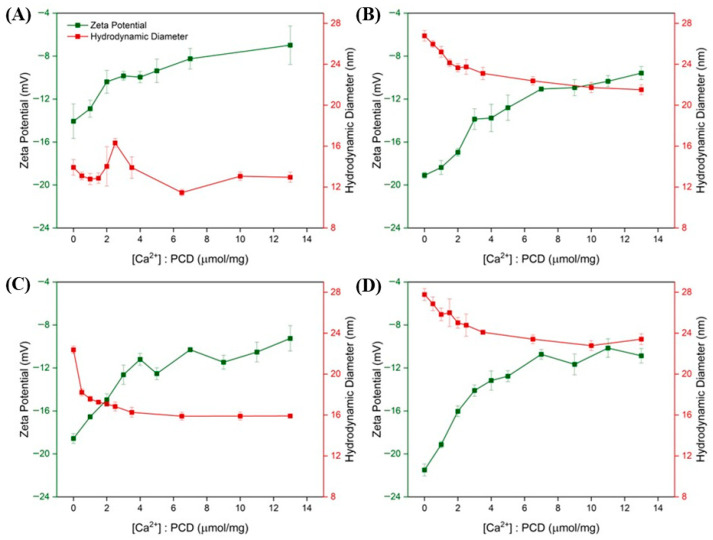
Changes in zeta potential (green lines) and hydrodynamic diameter (red lines) of the obtained PCDs as a function of Ca^2+^ concentration: (**A**) PCD 1:6, (**B**) PCD 1:9, (**C**) PCD 1:12, and (**D**) PCD 1:15.

Potentiometric titration profiles exhibited distinct potential shifts, suggesting an increased ability of the PCDs to complex Ca^2+^ with rising EDTA content. The amount of complexed Ca^2+^ was slightly lower than the theoretical 2:1 complexation ratio, indicating that most of available carboxyl groups are involved in effective ion binding. The correlation between zeta potential and Ca^2+^ complexation capacity suggests that carboxyl groups located primarily on the particle surface are engaged in chelation. The ability to bind divalent ions was further examined by monitoring zeta potential and hydrodynamic diameters on the course of titration with Ca^2+^ (Figure 4).

**Figure 5:**
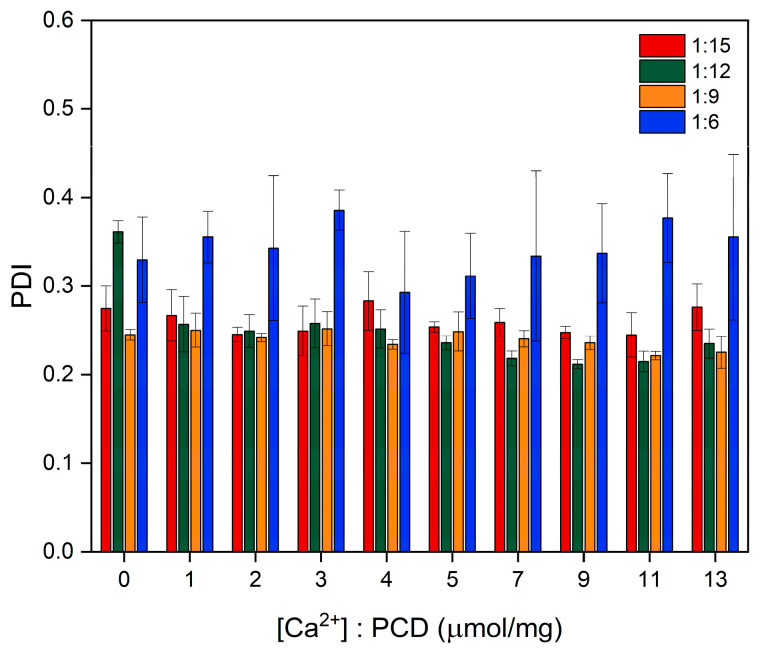
Effect of Ca^2+^ concentration on the PDI of PCDs with varying β-CD-to-crosslinker ratios.

The carboxyl groups derived from EDTA, which contribute to the negative surface charge of the PCDs, interact with Ca^2+^ upon their addition. This interaction results in the partial neutralization of surface charges, which explains the gradual increase in zeta potential. For all PCD variants, an initial increase in zeta potential was observed with rising Ca^2+^ concentration, followed by stabilization beyond a certain threshold of approximately 2.0 μM/mg of added Ca^2+^ relative to PCD. The remaining negative charge likely originates from uncomplexed carboxyl groups as well as other oxygen-containing functionalities within the network (e.g., esters, hydroxyls, glycosidic oxygens), which do not participate in Ca^2+^ coordination. These observations are consistent with the potentiometric titration data presented in Table 2. Differences between the initial zeta potential and the values observed at the end of titration correlate with the measured complexation capacity, further suggesting that chelation primarily involves surface groups. Furthermore, the reduction in the number of dissociated internal carboxyl groups may lead to decreased electrostatic repulsion within the polymer network. This effect may promote partial collapse of the polymer chains and an increase in crosslinking density through the formation of calcium-mediated ionic bridges, thereby explaining the observed tendency toward a decrease in particle size. At the later stage of Ca^2+^ addition, the particle hydrodynamic diameter stabilizes, likely as a result of the saturation of available calcium-binding sites and the lack of significant structural rearrangements. Because EDTA units are incorporated in a randomly crosslinked architecture, many carboxylate groups are not positioned to form the multidentate chelation motifs typical of free EDTA; therefore, saturation is reached before complete charge compensation. In contrast to the other variants, the PCD 1:6 formulation did not exhibit a clear trend toward decreasing hydrodynamic diameter. The assumed presence of a significantly lower number of carboxyl groups capable of interacting with Ca^2+^ in this variant may explain the less pronounced observable complexation effects. In PCD 1:6, the low EDTA content results in a network in which most EDTA units act as crosslinkers, leaving fewer free and sterically accessible carboxylate groups. In these crosslinking configurations, the remaining COOH/COO^−^ groups are often isolated rather than positioned adjacently, which limits their ability to participate in multidentate Ca^2+^ coordination. The absence of relevant changes in the PDI value indicated that, despite the increasing surface charge, the colloid remained stable and no particle aggregation was observed (Figure 5).

β-CD–based polymers have been similarly evaluated in simple ionic tests; for example, Trotta et al. reported characteristic metal-ion responses in pyromellitic dianhydride–crosslinked β-CD polymers, illustrating the broader relevance of ion-specific coordination in CD-based materials [11]. Although only Ca^2+^ was tested here, ions with different EDTA affinities (e.g., Mg^2+^, Zn^2+^, Fe^2+^/Fe^3+^) may induce distinct responses.

### 3.6. Interaction with Model Drugs

**Figure 6:**
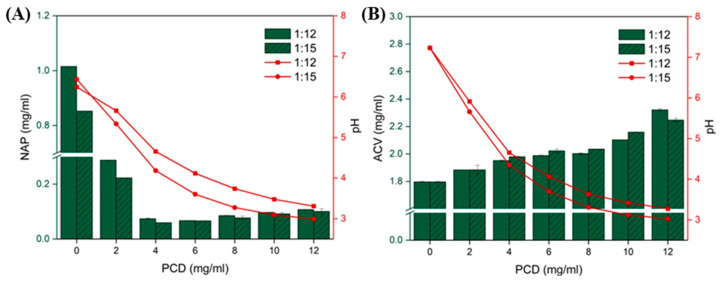
Changes in solubility of the model drug (**A**) NAP and (**B**) ACV as a function of PCD concentration (green bars), and the effect of increasing PCD concentration on the solution pH (red lines).

To evaluate the potential of the synthesized nanoparticles as drug delivery systems, the incorporation of two model active substances, NAP and ACV, into the polymeric network was assessed using an approach based on the Higuchi–Connors method. In the case of NAP, which is a weak acid, an initial decrease in drug solubility was observed with increasing concentrations of PCD (Figure 6A).

However, when the PCD concentration exceeded 6.0 mg/mL, a minor increase in solubility was noted, which was attributed to the incorporation of the active substance into the PCD matrix. The synthesized PCDs exhibit acidic properties, and increasing their concentration leads to a decrease in the overall pH of the solution, which in turn affects the ionization state of NAP, effectively reducing its solubility. When the pH drops below the pKa of NAP (pKa NAP = 4.15), the non-ionized form dominates, and the solubilizing effect of PCDs becomes visible. Nevertheless, this effect is weak in comparison to the solubility-decreasing impact of pH, which renders the investigated PCDs not suitable for acidic drug incorporation. Drug loading into polyanionic PCDs is inherently coupled to solution pH, because both the ionization state of the guest molecule and the charge state of the polymer network influence apparent solubility and complex stability. In the present study, drug-PCD interactions were evaluated in PBS to probe intrinsic affinity; however, the buffering capacity of PBS was insufficient to suppress the pH decrease induced by the acidic PCDs, which strongly affected the solubility of pH-sensitive drugs such as naproxen. Although drug loading could in principle be performed under alternative conditions that minimize pH effects (e.g., stronger buffering, pre-neutralized PCD solutions, or pH-adjusted loading media), such conditions would not necessarily reflect the environments encountered after administration. The pH and ionic composition experienced in vivo—such as neutral plasma, mildly acidic inflamed tissues, gastric fluid, or mucosal surfaces—will directly influence both the stability of drug–PCD inclusion complexes and the polymer’s ionization state. Thus, loading conditions that maximize drug uptake ex situ may differ from those governing complex stability and release in vivo.

For ACV, a linear increase in solubility was observed with increasing PCD concentration, with r^2^ of 0.8901 and 0.9696 for PCD 1:12 and PCD 1:15 variants, respectively (Figure 6B). According to the Higuchi and Connors model, this behavior corresponds to an A_L_-type phase solubility profile, indicating the formation of an inclusion complex between guest molecules (ACV) and the host (PCD) [20]. While A_L_ behavior corresponds to 1:1 inclusion in monomeric CDs, in crosslinked PCDs this represents an apparent 1:1 interaction model because the polymer contains a heterogeneous population of cavities with varying accessibility. The lower slope relative to free β-CD derivatives likely reflects partial steric hindrance within the crosslinked network. The calculated slopes, reflecting the mass of drug solubilized per mass of nanocarrier, were 0.0368 for PCD 1:12 and 0.0349 for PCD 1:15, showing similar values for both investigated variants. For comparison, this parameter was 0.1041 for sulfobutyl ether β-CD (Captisol^®^), while values of 0.1637 and 0.1984 were reported for hydroxypropyl-β-CD and unmodified β-CD, respectively [30,31,32]. These results indicate that the developed nanocarriers are capable of enhancing the solubility of ACV; however, the incorporation process is less effective than in formulations based exclusively on modified or unmodified β-CD. This can be explained by the fact that, in the investigated system, β-CDs capable of drug complexation represent only a fraction of the PCDs. Moreover, in polymeric systems, the efficiency of guest molecule inclusion may be lower due to crosslinking, which introduces additional steric groups bulk and rigidity that can distort the cavity environment, lowering the effective binding affinity. The size and shape of the cyclodextrin cavities may alter, hindering the formation of stable inclusion complexes and resulting in less effective encapsulation.

### 3.7. Formation of Polyelectrolyte Complexes

**Figure 7:**
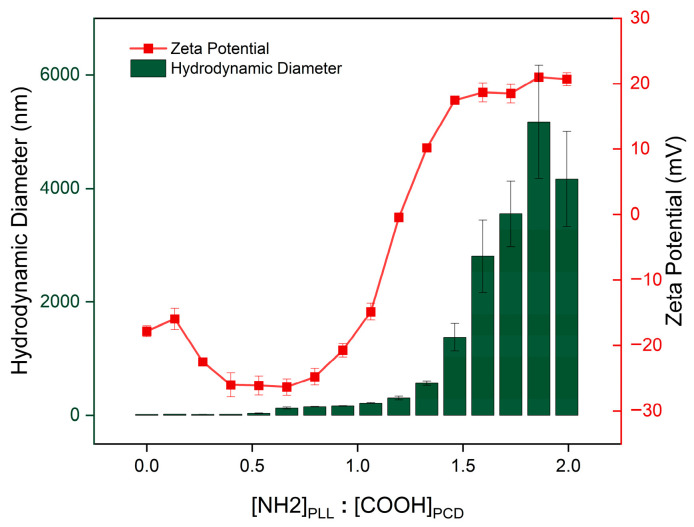
Variation in hydrodynamic diameter (green bars) and zeta potential (red line) following successive dosing of PLL to a PCD 1:12 solution.

The ability of PCDs to form polyelectrolyte complexes was evaluated using PLL as the polycation. An increase in turbidity of the PCD 1:12 solution was observed immediately after the addition of the first portion of PLL, indicating the onset of complex formation. Zeta potential analysis conducted following the sequential addition of PLL confirmed electrostatic interactions between the negatively charged PCD particles and the ionized –NH_2_ groups of the polycation. As the amount of PLL increased, a gradual rise in zeta potential was observed, following an initial plateau at negative values, corresponding to the neutralization of surface charge (Figure 7). A similar relationship was observed in the course of investigating the interaction between ε-PLL and β-CD sulfate [33].

At a [NH_2_]_PLL_:[COOH]_PCD_ ratio of 1.20, the zeta potential reached 0 mV, reflecting charge equivalence, and subsequently stabilized at approximately +20 mV. This stabilization indicates saturation of available binding points, and the predominance of positively charged –NH_2_ groups. A pronounced increase in hydrodynamic diameter was recorded above the [NH_2_]_PLL_:[COOH]_PCD_ ratio of ~1.30, further supporting the successful formation of PCD–PLL complexes. At this point, repulsive forces are minimized and PLL chains can bridge between multiple PCD particles, promoting cluster formation. With further PLL addition, the complexes became positively charged and partially restabilized, although the particle size remained elevated, consistent with irreversible aggregation and continued PLL-mediated bridging. Thus, the charge ratio dictates both complex charge and colloidal stability, with maximum aggregation occurring near charge neutralization. This may be attributed to interactions either between individual PCD–PLL complexes or between the complexes and excess unbound PLL molecules. It is worth noting that higher sample turbidity can lead to multiple light scattering and interfere with accurate particle size analysis [34].

Investigated PCDs demonstrated a remarkable multifunctionality, effectively engaging in interactions with small-molecule drugs, divalent ions, and oppositely charged polymers. This unique convergence of functionalities that positions them as promising platforms for advanced drug delivery and biomaterial design. Their cyclodextrin cavities enable inclusion-based solubilization of small molecules, while the EDTA moieties introduce an intrinsic ion sensitivity that can modulate particle size, charge, and as a result possibly drug retention in response to biologically relevant cations. Furthermore, their polyanionic nature allows controlled self-assembly with complementary polymers, opening opportunities for constructing multilayer coatings, hybrid hydrogels, or ion-responsive delivery matrices. Taken together, these combined features enable multifunctional behavior within a single carrier—drug complexation, ion-triggered structural transitions, and polyelectrolyte assembly—which can be leveraged to engineer smart, environmentally responsive therapeutic systems.

## 4. Conclusions

In this study, water-soluble PCDs were successfully synthesized and comprehensively characterized as multifunctional nanocarriers. The obtained PCDs exhibited a nanoscale size range, stable negative surface charge, and a high density of accessible carboxyl groups derived from EDTA. These features influenced not only the physicochemical characteristics of the PCDs but also their reactivity toward metal ions and oppositely charged polymers. Effective chelation of divalent cations such as Ca^2+^ resulted in measurable, ion-dependent changes in nanoparticle properties. The lowest crosslinker content resulted in particles with fewer reactive groups and reduced ion-binding capacity, whereas higher crosslinking levels led to enhanced functional performance. Phase solubility studies revealed the ability of PCDs to form inclusion complexes with ACV, while interactions with NAP highlighted limitations in loading weakly acidic drugs due to pH effects. Furthermore, experiments with PLL confirmed that PCDs can assemble into polyelectrolyte complexes, demonstrating their compatibility with polymeric systems. Collectively, these findings confirm the versatility of EDTA-PCDs as smart materials capable of ion complexation, drug encapsulation, and polymer assembly. Owing to their physicochemical properties, they are promising candidates for modulating drug release kinetics, enabling controlled and targeted therapeutic effects. This multifunctionality opens promising avenues for their application in biomedical fields, including ion-sensitive delivery systems, responsive coatings, and layer-by-layer systems.

## Figures and Tables

**Table 1:** Reaction yield and percentage elemental composition (C, N, O) of PCDs synthesized at various molar ratios of β-CD to crosslinking agent.

Variant	Reaction Yield (%)	N Content (%)	C Content (%)	O Content (%)
PCD 1:6	15.76	5.90	46.99	47.11
PCD 1:9	28.38	6.74	43.14	50.12
PCD 1:12	63.28	7.32	42.23	50.45
PCD 1:15	65.97	7.52	40.64	51.84

**Table 2:** Zeta potential, free carboxyl group content, and Ca^2+^ binding capacity of the synthesized PCDs.

Variant	Zeta Potential (mV)	COOH Content (μM/mg)	Ca^2+^ Binding (μM/mg)
PCD 1:6	−18.38 ± 2.45	4.096	1.601
PCD 1:9	−24.35 ± 1.03	4.641	2.112
PCD 1:12	−24.75 ± 0.33	5.150	2.137
PCD 1:15	−27.38 ± 2.51	5.502	2.319

## Data Availability

The original contributions presented in this study are included in the article/supplementary material. Further inquiries can be directed to the corresponding author..

## Supplementary Materials

The following supporting information can be downloaded at: https://www.mdpi.com/article/10.3390/ma19010207/s1. Figure S1. Dynamic light scattering (DLS) measurements of the PCD 1:6 sample: (A) intensity-weighted size distribution; (B) volume-weighted size distribution; (C) intensity autocorrelation function plotted as a function of delay time. Figure S2. Dynamic light scattering (DLS) measurements of the PCD 1:9 sample: (A) intensity-weighted size distribution; (B) volume-weighted size distribution; (C) intensity autocorrelation function plotted as a function of delay time. Figure S3. Dynamic light scattering (DLS) measurements of the PCD 1:12 sample: (A) intensity-weighted size distribution; (B) volume-weighted size distribution; (C) intensity autocorrelation function plotted as a function of delay time. Figure S4. Dynamic light scattering (DLS) measurements of the PCD 1:15 sample: (A) intensity-weighted size distribution; (B) volume-weighted size distribution; (C) intensity autocorrelation function plotted as a function of delay time.
