# Supplementary material for: Synthesis and Characterization of Water-Soluble EDTA-Crosslinked Poly-β-Cyclodextrins Serving as Ion-Complexing Drug Carriers
Source: Materials (Basel). 2026 Jan 5;19(1):207. doi: 10.3390/ma19010207 (PMC12786845; doi:10.3390/ma19010207)
Supplement: Supplementary file 1 [file materials-19-00207-s001.zip › materials-4025759-supplementary.pdf]

Supplementary Materials for Article

Synthesis and Characterization of Water-Soluble EDTA-Crosslinked Poly-β-Cyclodextrins Serving as Ion-Complexing Drug Carriers

Zuzanna Podgórniak [1], Witold Musiał [1,*], Michał J. Kulus [2], Dominika Łacny [1], Aleksandra Budnik [1] and Tomasz Urbaniak [1]

[1] Department of Physical Chemistry and Biophysics, Pharmaceutical Faculty, Wroclaw Medical University, Borowska 211, 50-556 Wrocław, Poland; zuzanna.podgorniak@student.umw.edu.pl (Z.P.); lacnydominika@gmail.com (D.Ł.); aleksandra.m.budnik@gmail.com (A.B.); tomasz.urbaniak@umw.edu.pl (T.U.)

[2] Division of Ultrastructural Research, Wroclaw Medical University, Chałubińskiego 6a, 50-368 Wrocław, Poland; michal.kulus@umw.edu.pl

* Correspondence: witold.musial@umw.edu.pl

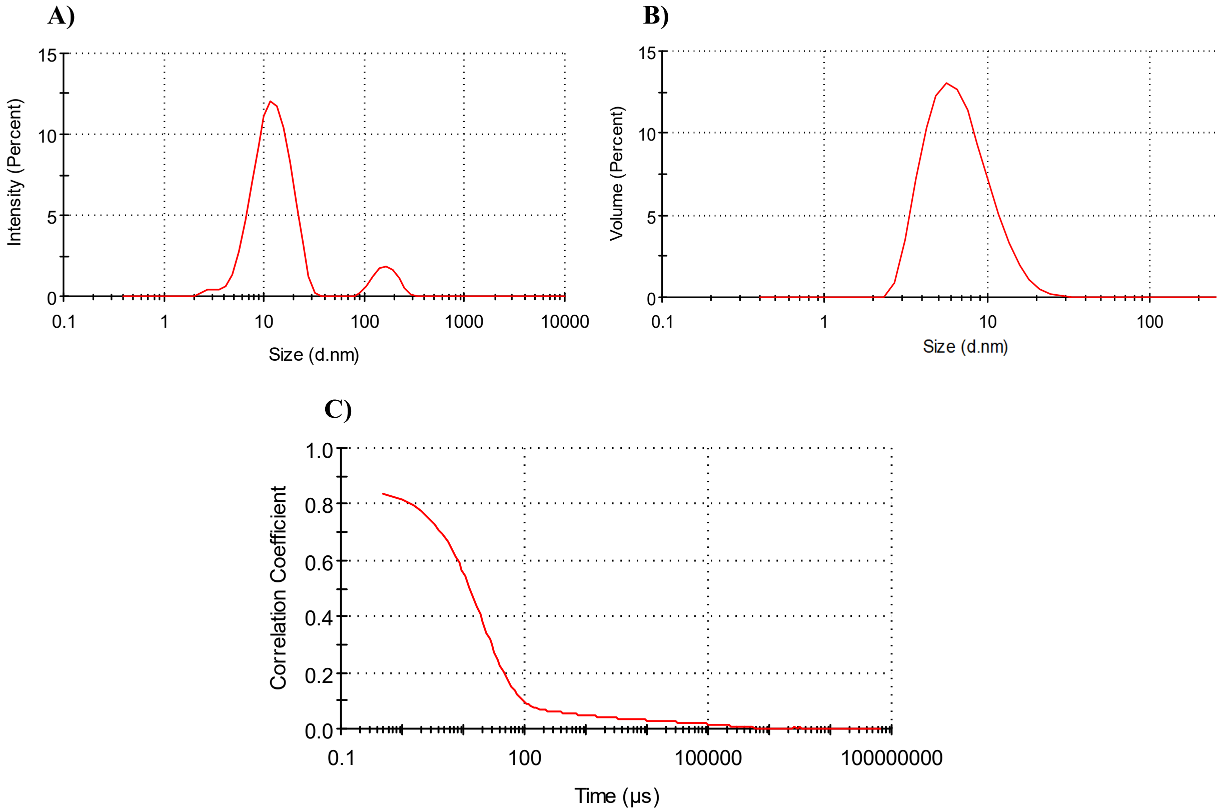

Figure S1. Dynamic light scattering (DLS) measurements of the PCD 1:6 sample: (A) intensity-weighted size distribution; (B) volume-weighted size distribution; (C) intensity autocorrelation function plotted as a function of delay time.

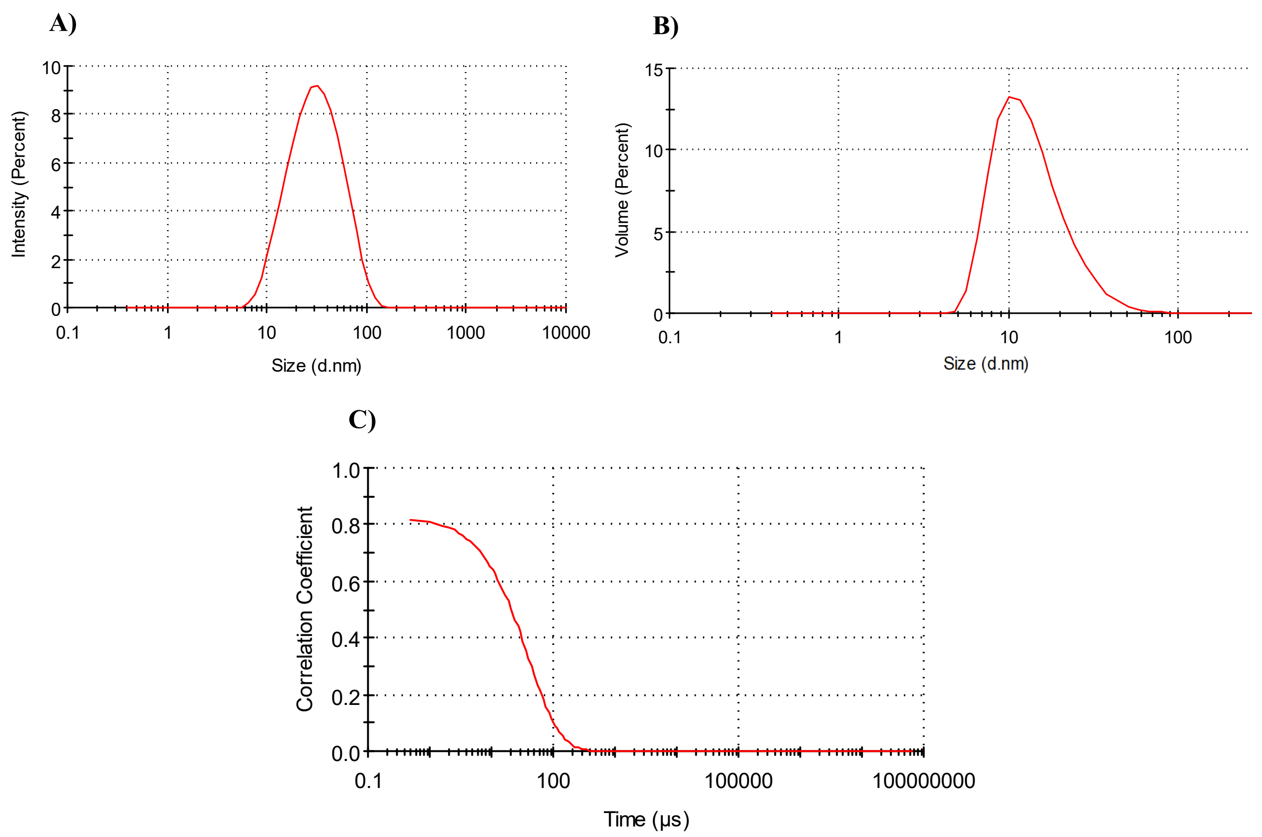

Figure S2. Dynamic light scattering (DLS) measurements of the PCD 1:9 sample: (A) intensity-weighted size distribution; (B) volume-weighted size distribution; (C) intensity autocorrelation function plotted as a function of delay time.

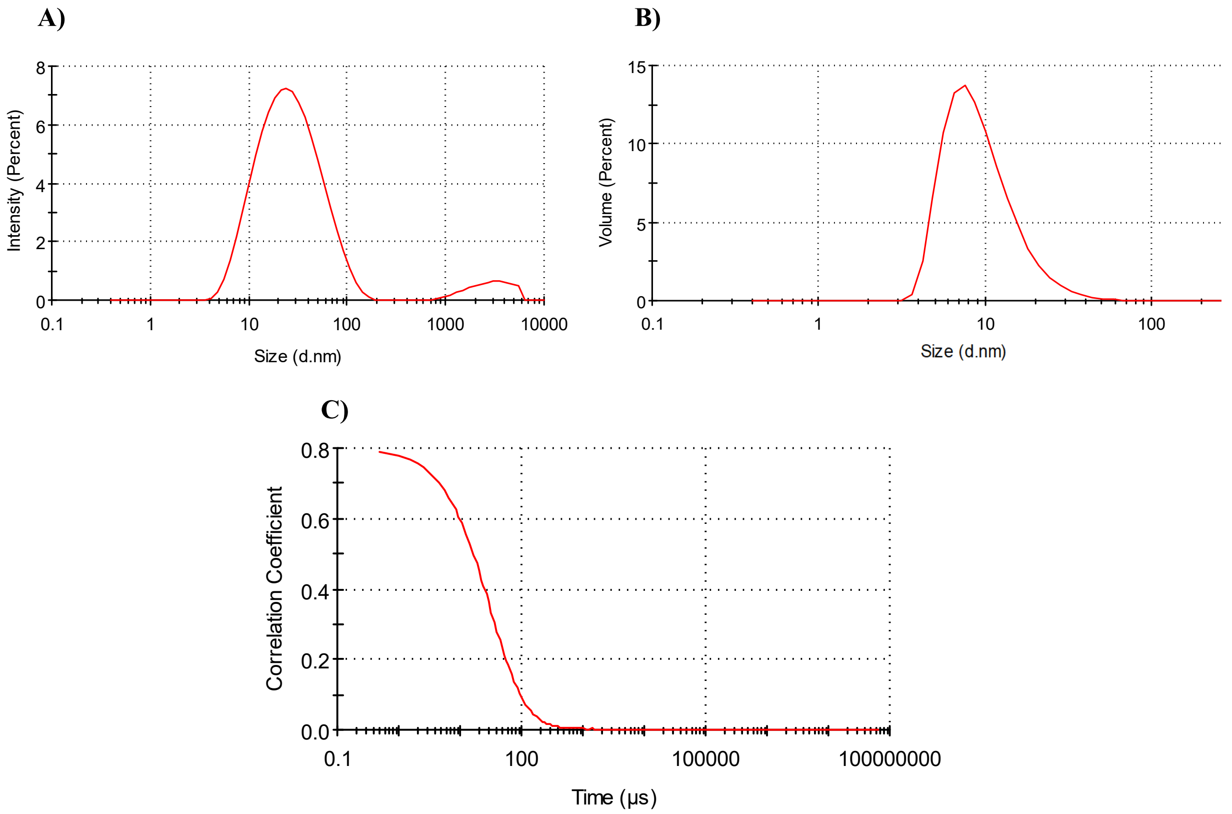

Figure S3. Dynamic light scattering (DLS) measurements of the PCD 1:12 sample: (A) intensity-weighted size distribution; (B) volume-weighted size distribution; (C) intensity autocorrelation function plotted as a function of delay time.

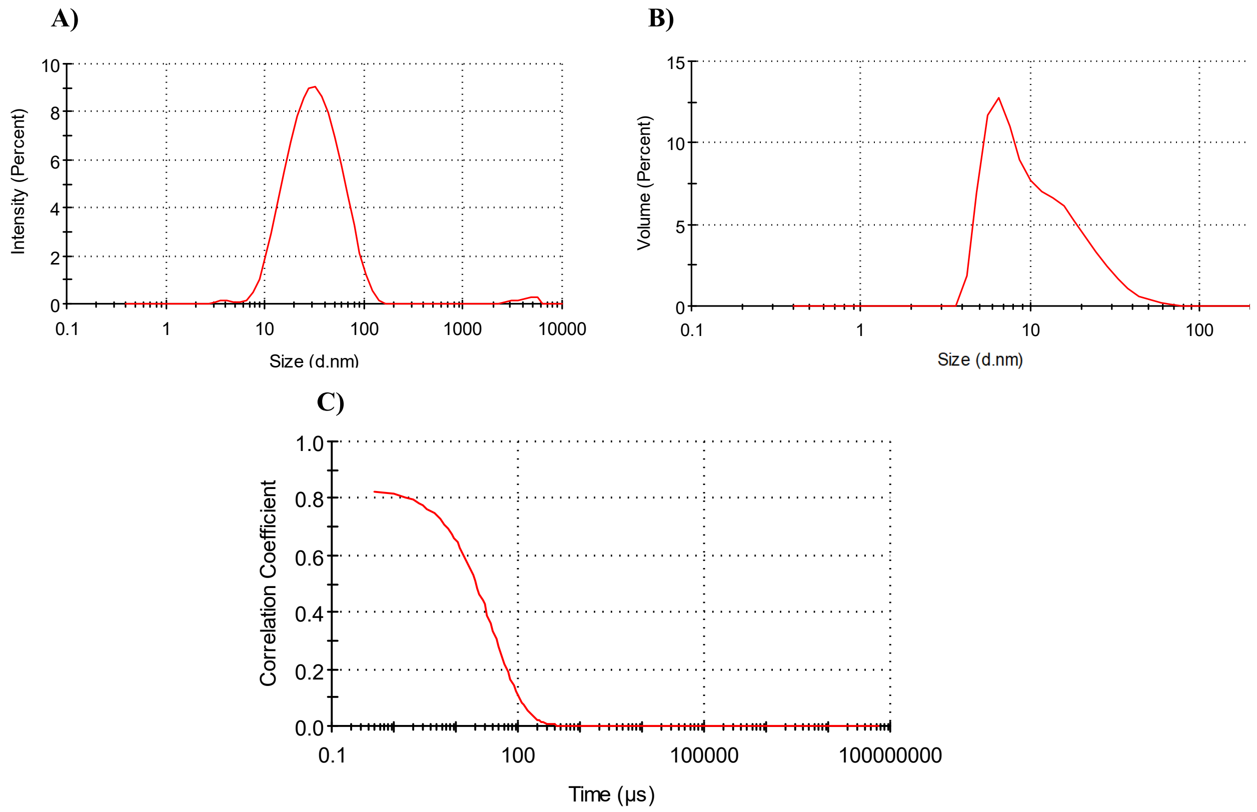

Figure S4. Dynamic light scattering (DLS) measurements of the PCD 1:15 sample: (A) intensity-weighted size distribution; (B) volume-weighted size distribution; (C) intensity autocorrelation function plotted as a function of delay time.
